# The Effect of Guided Reflection on Test Anxiety in Nursing Students

**DOI:** 10.5812/nms.11119

**Published:** 2013-09-15

**Authors:** Farkhondeh Sharif, Razieh Dehbozorgi, Arash Mani, Mehrdad Vossoughi, Pouran Tavakoli

**Affiliations:** 1 Community Based Psychiatric Care Research Center, Department of Mental Health and Psychiatric Nursing, School of Nursing and Midwifery, Shiraz University of Medical Sciences, Shiraz, IR Iran; 2 Department of Psychiatry, Faculty of Medicine, Shiraz University of Medical Sciences, Shiraz, IR Iran; 3 Department of Dental Public Health, School of Dentistry, Shiraz University of Medical Sciences, Shiraz, IR Iran

**Keywords:** Anxiety, Guided reflection, Nursing students, Test

## Abstract

**Background:**

Anxiety disorders are common and test stress affects many students. Guided reflection is a new and effective method for reducing stress.

**Objectives:**

To assess the effect of guided reflection on test anxiety in second and third-year nursing students of Fatima Nursing and Midwifery College, Shiraz University of Medical Sciences, Shiraz, Iran.

**Materials and Method:**

This study was designed to assess the effect of guided reflection on test anxiety among nursing students of second and third year of education in Faculty of Nursing and Midwifery, Shiraz University of Medical Sciences, Shiraz, Iran. Data was collected using demographic data questionnaires and the Sarason and Abolghasemi test anxiety scale. Based on the latter questionnaire, of the 147 participants, 100 had test anxiety with scores ranging from 13-63 (mild-severe anxiety), of which 80 students were randomly selected and divided into case and control groups. We used Johns’s 9-stage guided reflection model through Q and A, lecture, and discussion. The case group participated in a 2-day guided reflection workshop for six hours each day. The control group received no intervention. Sarason and Abolghasemi’s test anxiety questionnaire was completed by the students at the beginning of the first session, immediately after and three months after intervention.

**Results:**

The test anxiety mean scores were 35.34 ± 9.50 and 35.47 ± 10.66 before the intervention in the control and intervention groups respectively. No significant difference was found between the two groups with respect to socio-demographic characteristics. The Mean ± SD of test anxiety scores increased to 36.48 ± 9.34 three months after the intervention in the control group (P = 0.1). However, in the intervention group, the Mean ± SD test anxiety scores reduced immediately after (16.31 ± 8.61) and three months after (27.72 ± 10.09) the intervention, compared to before the intervention (35.47 ± 10.66) (P < 0.001, paired T-test).

**Conclusions:**

Guided reflection is effective in reducing test anxiety in nursing students. This method can be used for reducing test anxiety and increasing learning and academic progress among students.

## 1. Background

Anxiety is an undesirable and vague feeling which is felt when a person anticipates or predicts uncertain danger (1). People of all eras with different cultures have at times experienced such a feeling. On the other hand, we also have a type of negative morbid anxiety which is the root of many physical and mental disorders and unjustified panic and fear (2).

In recent decades, many studies have been performed on anxiety and related fields showing that anxiety disorders are quite prevalent in the society. One of these orders is test anxiety (3). Test anxiety is an important educational problem affecting millions of students worldwide, and is an undesirable emotional reaction to the situation to be evaluated. Test anxiety is a threat to people's mental health and negatively affects their efficiency, talent, personality, and social identity (4). The American Psychological Association defined test anxiety as a form of anxiety which leads to increased stress, negative self-perception, increased chance of cheating, and reduced motivation and exam performance related to the fear of being evaluated (5).

Test results have an immense impact on various aspects of people's lives. Students face more exams as they excel in their studies, as well as higher expectations from their parents and the educational system. Such factors increase test stress (6). In one third of students test stress leads to mental disorders such as anxiety and depression (7). Stressors include test scores, exams, long study hours, career adjustment, and familial commitments and can reduce educational performance, concentration, memory, and problem solving skills (8). Nursing students experience higher stress and acute stress most often tends to chronic stress in these students over time (6). The American Holistic Nursing Association reported that students who cannot adapt with stress experience symptoms such as depression, anxiety, sleep disorder, headaches, gastrointestinal disorders such as irritable bowel syndrome, memory loss, and judgmental problems in difficult situations which reduce their communicational skills, self-actualization skills, and sympathy (9).

Some methods have been previously proposed to reduce test stress in students such as assertiveness and stress management training, effect of metacognitive and self-efficacy beliefs, Islamic coping skills training, and cognitive-behavioral therapy (10-15). One of the most effective treatment methods is the reflection method. This method assists students in related emotional recognition (6). The first and most fundamental theory of reflection is that of Deweys. He emphasized that the models function is to transform the conflict, doubtful, and vague situation to a clearer, and more coherent and harmonic condition. He believed that reflection encourages individuals to search and find principles that would solve their problems. Moreover, he understood the importance of previous experiences for reflection and believed that ideas and suggestions are related to an individual's unique experiences (16).

Reflection is the method with which a person refers to his or her previous experience and recollects that experience, put it forth once again, and reevaluates it (17). Beggs writes: Boyd and Fales presented a very common definition for learning through reflection. They stated that learning through reflection is the process with which people can explore and examine internal issues. Reflection begins with experimentation and creates a changed conceptual perspective. Reflection is useful and effective for those who strive to reach new evaluations and inferences (6). Some studies have used tools such as portfolios and educational achievement records for reflection (17), while others have considered the view points and experiences of students and teachers. Jensen and Joy found that many students are amateurs in reflective exercises and as a result cannot completely benefit from this method to gain academic success (18). John's guided reflection method which was presented in 2009 can be used to solve this problem. The guide assists the student in expressing feelings related to their experiences. By reflecting their test-related experiences, students with test stress discover thoughts that trigger stress and anxiety. Identifying the source of stress helps students to select suitable management techniques for their holistic self-care practices (6). Beggs writes: Beck and Srivastava believe that holistic self-care programs assist students reduce their educational distress and increase their adaptation capabilities (6). Considering the high rate of test stress among nursing students (6) and the important association between anxiety and learning (19) it is important to investigate the effect of guided reflection on test anxiety however, there is a lack of study in this regard.

## 2. Objective

This study aimed to assess the effect of guided reflection on test anxiety in second and third-year nursing students of Fatima Nursing and Midwifery College, Shiraz University of Medical Sciences, Shiraz, Iran.

## 3. Materials and Methods

**Figure 1.**
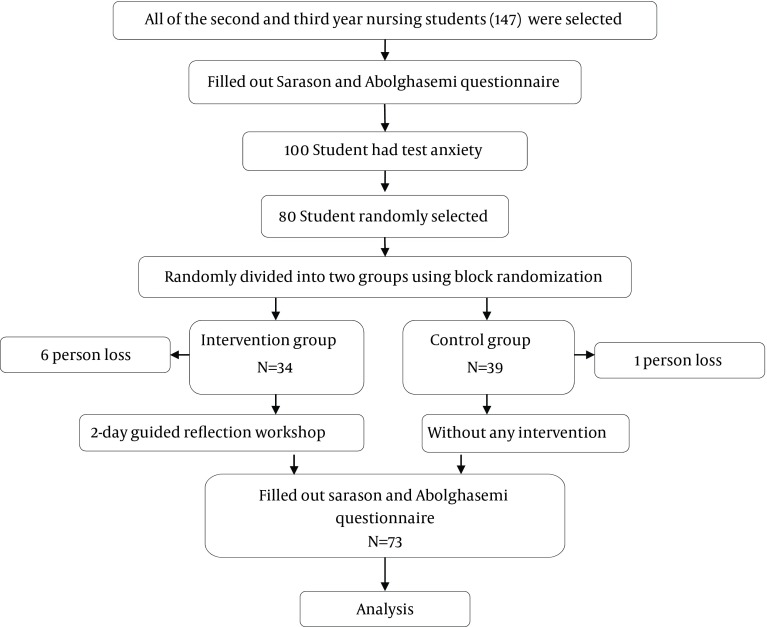
Consort Flow Diagram of the Study

In this before-after interventional study, initially all of the second and third year nursing students (n = 147) studying at Shiraz University of Medical Sciences, Shiraz, southern Iran, during 2012 were selected. All students completed the Sarason and Abolghasemi questionnaire. 100 students who had test anxiety with scores ranging from 13-63 (mild-severe anxiety) and fulfilled the inclusion criteria were enrolled to participate in the study. Each student was given a number and finally 80 students were randomly selected from those who enrolled, and divided into two groups of intervention and control using block randomization, then one person lost from the control group and 6 persons from intervention group due to unwillingness to participate in this study (Figure 1). Other inclusion criteria were: being second and third-year students, willing to participate in the study, completing the consent form, and having anxiety according to the Sarason and Abolghasemi questionnaires. The exclusion criteria were: lack of participation in guided reflection sessions, having mental disorders such as anxiety disorders and depression based on self-report forms. Data was collected using demographic data questionnaires and the Sarason and Abolghasemi test anxiety scale by the researcher during the March exams and those with mild-severe anxiety were included. The demographic data questionnaire consisted of information regarding the students’ age, gender, education level (second or third academic year), economic status (less than 2000000 to above 8000000 Rials), history of mental disorders (such as depression and anxiety), number of siblings, period of anxiety during the months, and whether their parents were alive or deceased. 

Sarason and Abolghasemi questionnaire consisted of 25 multiple choice questions rated on a scale of 0-3 (never, seldom, sometimes, and often). The minimum and maximum scores are 0 and 75. A higher score shows higher anxiety. Therefore, participants who had a score of <12 were considered as not having any anxiety, 13-37 having mild anxiety, 38-63 having moderate anxiety, and >63 as having severe anxiety. The reliability and validity of this questionnaire have been previously evaluated (88% test-retest validity, 95% internal consistency, and 72% criterion validity) (20). Each person was given a number and randomly selected, of which 80 students were randomly selected 

Johns’s 9-stage guided reflection model which consists of preparation, pick-up, listening, clarifying, understanding, options, taking action, empowering and wrap up through Q and A, lecture, and discussion for guidance and Johns’s 5-stage model of structured reflection for students were used in this study. The intervention group participated in a 2-day guided reflection workshop for six hours each day. The model consists of five cue questions related to description of experience, reflection, influencing factors, approaches for dealing with the situation better, and learning. Since guided reflection is a new method and because of the lack of knowledge students had about this method, the researcher gave her phone number and address to the students so they could be able to ask about reflection on their experiences.

Sarason and Abolghasemi’s test anxiety questionnaire was completed by the students at the beginning of the first session, immediately after and three months after intervention. The control group received no intervention. However, the participants in the control group received related educational pamphlets after the study.

### 3.1. Data Analysis

Data was analyzed using SPSS software, version 15. Descriptive data were presented as frequency, percentage, mean and standard deviation. To compare test anxiety scores before, immediately after and three months after the intervention in the intervention group repeated measurement test was used, and to compare this scores before and three months after intervention, T-test was used.

### 3.2. Ethical Considerations

Ethical considerations used in this study were as follows: Providing a letter of introduction from Faculty of Nursing and Midwifery, completing an informed consent form, confidentiality of student information, providing a certificate for the intervention group and educational materials for the control group. Also the study was approved by the Ethics Committee of Shiraz University of Medical Sciences.

## 4. Results

In total, 54 (67.9%) women and 26 (32.1%) men participated in this study. The (Mean ± SD) test anxiety scores were 35.34 ± 9.50 and 35.47 ± 10.66 before the intervention in the control and intervention groups respectively (P = 0.950). Moreover, no significant difference was found between the two groups with respect to socio-demographic characteristics such as age, gender, economic status, mental health, and grade point average (Table 1). The Mean ± SD anxiety scores before the intervention were 36.91 ± 11.50 and 37.52 ± 9.34 in women and men, respectively (P = 0.811). The Mean ± SD test anxiety scores increased from 35.34 ± 9.50 before the intervention to 36.48 ± 9.34 three months after the intervention (P = 0.140) in the control group. However, in the intervention group, the Mean ± SD test anxiety scores reduced immediately after (16.31 ± 8.61) and three months after (27.72 ± 10.09) the intervention, compared to before the intervention (34.88 ± 9.45) (in both groups P < 0.001), (Table 2). 

**Table 1.** Comparison of the Main Variables in the Intervention and Control Groups

Variables	Control, (n = 39)	Intervention, (n = 34)	P value
**Gender**			
Male	11	15	
Female	29	26	
**Age**	1.41 ± 21.06	1.51 ± 20.97	0.380
**Grade point average**	1.23 ± 14.90	1.29 ± 15.07	0.560
**Mental health**	14.7 ± 62.19	11.8 ± 64.47	0.450
**Test anxiety score**	9.50 ± 35.34	10.66 ± 35.47	0.950

The quantitative variables were reported as (Mean ± SD) in this table. The Sex was reported as the ratio of number of male to female.

**Table 2.** Mean ± SD anxiety Scores

	Case, Mean±SD	Control, Mean ± SD
**Before intervention**	34.88 ± 9.45	35.34 ± 9.50
**Immediately after intervention**	16.31 ± 8.61	-
**Three months after intervention**	27.72 ± 10.09^b^	36.48 ± 9.34
**P value**	<0.001	0.140

Different letters in superscript following values indicate pairwise statistical significance between the measurements in the intervention group.

## 5. Discussion

In this study, guided reflection significantly improved test anxiety in the intervention group immediately and three months after the intervention, which is consistent with another recent similar study (6). In the mentioned study, the researchers found that test anxiety scores increased in the intervention group three months after their intervention, which could be related to the effect of time and forgetting the content of the workshops. Some studies have evaluated the effect of reflection on clinical experiences in students and we found few studies that had evaluated the effect of this method on reducing anxiety or test anxiety. Studies on reflection have shown its positive effect on different variables such as critical thinking in students and nurses, nurses’ thinking style, clinical training, and daily portfolios (17, 21-23).

In another study in Iran, the researchers evaluated the effect of reflection on clinical performance portfolios on critical thinking skills on 42 nursing students. They found that a 2-week reflection was effective on five critical thinking skills (23). In a study on the effect of reflection on critical thinking, the researchers found that reflection develops and enhances critical thinking and the tendency to think critically among students and teachers (22). Consistently, Moattari et al. also found that reflection was effective on the students’ inductive reasoning as well as their total critical thinking score (24). Selby has also quoted a study in which Richardson and Maltby studied reflection portfolios as a tool for enhancing learning and self-evaluation. They reported that the majority of students had engaged in conceptual and theoretical reflection which is the highest level of reflection (25). Lowe and Kerr used an experimental design to assess learning in two matched groups of students. They used reflective teaching methods for one group and the conventional educational method for the other, and eventually evaluated their learning. The results did not show any significant difference between the two groups. They concluded that the intervention group had learned the reflection method compared to the other group and suggested further studies to be performed due to high learning potential in this group (26).

Guided reflection is effective in reducing test anxiety in nursing students. This method can be used for reducing test anxiety and increasing learning and academic progress among students. Possibility of information exchange between control and intervention groups was one of the limitations of our study. To prevent this problem, the intervention program was held outside the students' classroom hours. 

Providing a guided reflection plan based on the results of the study to students with test anxiety can improve their academic performance and change their negative attitude to a positive attitude.
